# Promoting seamless transfer-of-care for stroke patients in primary care: development of the integrated care pathway for post stroke services

**DOI:** 10.1186/1471-2458-14-S1-O9

**Published:** 2014-01-29

**Authors:** Aznida Firzah Abdul Aziz, Noor Azah Abd Aziz, Nor Azlin Mohd Nordin, Saperi Sulong, Syed Mohamed Aljunid

**Affiliations:** 1 UNU IIGH, Universiti Kebangsaan Malaysia, UKM Medical Centre Complex, Jalan Yaacob Latif, 56000 Cheras, Kuala Lumpur, Malaysia; 2 Universiti Kebangsaan Malaysia, Jalan Yaacob Latif, Bandar Tun Razak, 56000 Cheras, Kuala Lumpur, Malaysia; 3 United Nations University International Institute for Global Health (UNU-IIGH), 56000 Cheras, Kuala Lumpur, Malaysia

## Background

Post-stroke care after hospital discharge suffers from lack of intersectoral collaboration within the public health sectors. Hence, primary care remains the only option in managing stroke patients in underserved areas in Malaysia. This study aimed to identify the areas, which can be better coordinated to deliver optimal post-stroke care in community setting. A seamless transfer of care model known as integrated Care Pathway for Post Stroke patients (iCaPPS) was designed to address this issue.

## Materials and methods

Expert panel discussions comprising of family physicians, neurologists, rehabilitation physicians and therapists, and nurse managers from both Ministry of Health and the academia were conducted. Modified Delphi technique was employed to resolve practice variations through additional literature support. Care algorithms were designed around existing work schedules and available resources at public health centres.

## Results

Identified areas for coordinated transfer of care include: identification of patient criteria suitable for long-term stroke management at primary care facilities, information required at transfer of care, stroke risk factors treatment targets, screening for stroke complications and rehabilitation guide for primary care team. Care algorithm including appropriate tools were summarised to identify patients requiring further multidisciplinary rehabilitation interventions i.e. assessment for those uninitiated or missed out on rehabilitation and leisure intervention for those indicated, screening for swallowing disorders as well as mental health disorders (i.e. depression and dementia).

## Conclusions

The iCaPPS would facilitate coordinated primary care-led post-stroke management for patients residing at home in the community, hence promoting better collaboration within public health sectors. Clinical outcomes and cost effectiveness of iCaPPS can be evaluated for benefit of stakeholders and stroke survivors.

